# ‘Nebbiolo’ genome assembly allows surveying the occurrence and functional implications of genomic structural variations in grapevines (*Vitis vinifera* L.)

**DOI:** 10.1186/s12864-022-08389-9

**Published:** 2022-02-24

**Authors:** Simone Maestri, Giorgio Gambino, Giulia Lopatriello, Andrea Minio, Irene Perrone, Emanuela Cosentino, Barbara Giovannone, Luca Marcolungo, Massimiliano Alfano, Stephane Rombauts, Dario Cantu, Marzia Rossato, Massimo Delledonne, Luciano Calderón

**Affiliations:** 1 Department of Biotechnology, University of Verona, Strada Le Grazie 15, 37134, Verona, Italy; 2 Institute for Sustainable Plant Protection, National Research Council (IPSP-CNR), Strada delle Cacce 73, 10135 Torino, Italy; 3 Department of Viticulture & Enology, University of California Davis, 595 Hilgard Lane, Davis, CA 95616 USA; 4 Department of Bioinformatics and Systems Biology, Ghent University, Technologiepark 927, B-9052 Gent, Belgium; 5 VIB Center for Plant Systems Biology, 9052 Gent, Belgium; 6 Instituto de Biología Agrícola de Mendoza (IBAM, CONICET-UNCuyo), Almirante Brown 500, M5528AHB. Chacras de Coria, Mendoza, Argentina

## Abstract

**Background:**

‘Nebbiolo’ is a grapevine cultivar typical of north-western Italy, appreciated for producing high-quality red wines. Grapevine cultivars are characterized by possessing highly heterozygous genomes, including a great incidence of genomic rearrangements larger than 50 bp, so called structural variations (SVs). Even though abundant, SVs are an under-explored source of genetic variation mainly due to methodological limitations at their detection.

**Results:**

We employed a multiple platform approach to produce long-range genomic data for two different ‘Nebbiolo’ clones, namely: optical mapping, long-reads and linked-reads. We performed a haplotype-resolved de novo assembly for cultivar ‘Nebbiolo’ (clone CVT 71) and used an *ab-initio* strategy to annotate it. The annotated assembly enhanced our ability to detect SVs, enabling the study of genomic regions not present in the grapevines’ reference genome and accounting for their functional implications. We performed variant calling analyses at three different organizational levels: i) between haplotypes of clone CVT 71 (primary assembly vs haplotigs), ii) between ‘Nebbiolo’ and ‘Cabernet Sauvignon’ assemblies and iii) between clones CVT 71 and CVT 185, representing different ‘Nebbiolo’ biotypes. The cumulative size of non-redundant merged SVs indicated a total of 79.6 Mbp for the first comparison and 136.1 Mbp for the second one, while no SVs were detected for the third comparison. Interestingly, SVs differentiating cultivars and haplotypes affected similar numbers of coding genes.

**Conclusions:**

Our results suggest that SVs accumulation rate and their functional implications in ‘Nebbiolo’ genome are highly-dependent on the organizational level under study. SVs are abundant when comparing ‘Nebbiolo’ to a different cultivar or the two haplotypes of the same individual, while they turned absent between the two analysed clones.

**Supplementary Information:**

The online version contains supplementary material available at 10.1186/s12864-022-08389-9.

## Background

*Vitis vinifera* was the fourth crop for which a reference genome was made available [1]. Because of the high heterozygosity levels, inherent to grapevine cultivars, a nearly homozygous genotype derived from ‘Pinot noir’ (PN40024) was sequenced [1] in order to ease the assembly process. Although technically convenient, homozygous materials have provided several limitations in understanding grapevines genomic complexity [2, 3]. At the same time, it has been observed that a single reference is not enough to capture the genetic landscape of a species, hence the concept of pan-genomes has been introduced, including core genomic features common to all individuals and a dispensable genome composed of genomic features not shared by all individuals [4]. The pan-genome of multiple plant species such as cabbage [5], sunflower [6] and poplar [7] has been characterized, reinforcing the need for studying the whole genome assemblies of multiple individuals of the same species. Grapevine is no exception, and the production of novel genome and transcriptome assemblies are aiding in unveiling the genetic bases of each cultivar’s particularities [2, 8–10]. In fact, every time a new grapevine genome is annotated novel genes and isoforms of genes are predicted [11].

Grapevines are clonally propagated, aiming to preserve the phenotypic traits that provide typicity to each cultivar [12]. Therefore, as a clonal crop, grapevines offer a suitable biological model to study structural variations (SVs). Moreover, the absence of out-crossing provides a proper genomic environment for SVs to accumulate as heterozygous recessives, making grapevines’ genomes highly unbalanced [2]. Therefore, characterizing SVs in grapevines is a fundamental task, because it has been shown that SVs have great impact on phenotypic traits of productive interest, such as colour [13] resistance to pathogens [14] and flower features associated to the sex determination [15].

SVs are defined as genomic rearrangements of at least 50 bp in size [16] and include deletions (DELs), insertions (INSs), duplications (DUPs) and translocations (TRA) [17]. Even though the characterization of SVs is considered essential for understanding the genome complexity, progress on their study is notably lagging behind the thorough comprehension achieved, for example, for single nucleotide polymorphisms (SNPs) [18, 19]. In fact, widely adopted short-read platforms provide only indirect evidence to infer the presence of SVs [20] resulting in a high rate of SVs miscalls, especially in repetitive regions that short reads cannot resolve properly [17]. However, a wide variety of long-range genomic platforms have recently emerged, including long-reads and linked-reads (i.e. short-reads confined within a relatively long DNA fragment), among many others [18]. These new platforms have allowed sequencing longer molecules, which helped overcoming alignment issues in repetitive regions, thus enabling the direct detection of SVs [18].

At the same time, long-range genomic platforms have also contributed to improve the de novo genome assembly process, yielding highly contiguous assemblies up to chromosome-scale level. In particular, high-quality diploid genome assemblies for highly heterozygous crops have been obtained, such as: *Brassica rapa*, *Brassica oleracea* [21], *Manihot esculenta* [22], as well as several *V. vinifera* L. cultivars [2, 15, 23–25]. These assemblies offer a smoother starting point to identify novel features, previously hidden in collapsed and fragmented genome assemblies [15, 22]. Integration of multiple platforms is a common practice to obtain high-quality genome assemblies [2, 3, 21, 22, 26]. However, fewer studies have simultaneously compared the contribution of multiple platforms and methodologies to detect SVs, especially in plants [19, 27, 28].

‘Nebbiolo’ is a grapevine (*V. vinifera* L.) cultivar appreciated for high-quality red wines production (e.g. Barolo and Barbaresco); it has been cultivated since the thirteenth century in north-western Italy, across the Piedmont, Aosta Valley and Lombardy regions [10]. A recent study based on short-reads genomic data identified diagnostic single nucleotide variants (SNVs) among three clones, representing the ‘Nebbiolo’ biotypes “Michet” (CVT 71), “Lampia” (CVT 185) and “Picoutener” (CVT 423), which are associated to different cultivation areas [10]. The availability of only short-reads data contributed to make the de novo genome assembly and the precise identification of SVs in ‘Nebbiolo’ not possible at the time [10].

In this work we assembled and annotated a genome for cultivar ‘Nebbiolo’ (clone CVT 71), that was used as base-line information to compare ‘Nebbiolo’ with four other cultivars at the functional level. At the same time, the obtained assembly was employed to survey the occurrence of SVs and their functional implications at three different organizational levels: haplotypes, clones and cultivars. In particular, we compared the SVs occurrence between the two assembled haplotypes of clone CVT 71. We also surveyed the presence of differentially occurring SVs between clones, by comparing ‘Nebbiolo’ biotypes “Michet” (CVT 71) and “Lampia” (CVT 185). Finally, we investigated SVs differentiating cultivars ‘Nebbiolo’ and ‘Cabernet Sauvignon’, exploiting an available assembly [24]. This was performed by means of three alternative methodological approaches, based on: long-reads, linked-reads, and genome-to-genome alignment. As a complementary objective, we evaluated the relative performance of the different methodological approaches employed here at detecting SVs. The improved technological capability allowed us to obtain a high-quality assembly for ‘Nebbiolo’, highlighting that SVs accumulation rate and functional impact strongly depend on the organizational level under study.

### Results

## ‘Nebbiolo’ de novo genome assembly

We performed a de novo genome assembly for ‘Nebbiolo’, using clone CVT 71 as biological material and integrated PacBio (long-reads), Bionano Genomics (optical mapping) and Illumina (short-reads) data. First, we assembled PacBio long-reads de novo, yielding 875 primary contigs (767 Mbp) and 3,911 alternative haplotypes (i.e. haplotigs) (405 Mbp), with a diploid preliminary assembly N50 = 1.2 Mbp. This assembly was polished for sequence errors with PacBio and Illumina reads. Then, we generated 157 Gbp of Bionano single-molecule maps and assembled them de novo into 969 optical consensus maps, totalling 1.1 Gbp (N50 = 1.5 Mbp). The consensus maps were used to anchor and scaffold the polished preliminary assembly, to produce a more contiguous hybrid assembly. The hybrid assembly consisted of 978 anchored sequences (816 Mbp) and 4,000 not-anchored sequences (356 Mbp), adding 1.2 Gbp (N50 = 2.5 Mbp), which is twice as big as the expected haploid genome size for grapevines (Table S1). Therefore, we separated the two haplotypes to reduce redundancy, which may hamper SVs detection by decreasing mapping quality of reads aligned to homologous regions. This process, based on the identification of homologous sequences with diploid read-coverage resulted in two assemblies, which we refer to as ‘Nebbiolo primary assembly’ and ‘Nebbiolo alternative haplotypes’. After obtaining the haplotyped subgenomes, we exploited the increased ability to map reads and performed two additional rounds of polishing using the Illumina reads. The primary assembly was 561 Mbp in size, consisted of 230 sequences (N50 = 5.4 Mbp) and included 94.8% of complete universal single-copy orthologue (BUSCO) genes. The alternative haplotypes were 534 Mbp in size, consisted of 1,987 sequences (N50 = 1.2 Mbp) and included 77.9% of complete BUSCO genes (Table 1). A total of 2,115 contigs, with 107 Mbp in length (N50 = 0.06 Mbp), were discarded as they were identified as assembly artefacts, based on either too high or too low read-coverage depth.

**Table 1:** Contiguity and completeness statistics for ‘Nebbiolo’ CVT 71 genome assemblies

	**‘Nebbiolo’ primary assembly**	**‘Nebbiolo’ alternative haplotypes**
Total assembly length (Mbp)	560.26	533.95
Assembly N50 (Mbp)	5.37	1.18
Total scaffolds length (Mbp)	487.09	225.01
Number of scaffolds	109	107
Number of gaps	384	247
Gaps size (Mbp)	20.51	8.68
Contigs in scaffolds	493	354
Remaining contigs	121	1,880
Remaining contigs total length (Mbp)	73.17	308.94
BUSCO statistics	C:94.8%, F:2.0%, M:3.2%	C:77.9%, F:1.9%, M:20.2%
Num. genes	35,038	32,865
Perc. repetitive content	52.9%	56.5%

For BUSCO statistics, ‘C’ refers to gene completeness, ‘F’ to fragmented genes, and ‘M’ to missing genes. ‘Nebbiolo’ primary assembly and alternative haplotypes refer to the genome assemblies obtained after haplotypic separation

### Genome annotation and comparative functional enrichment analysis

**Fig. 1:**
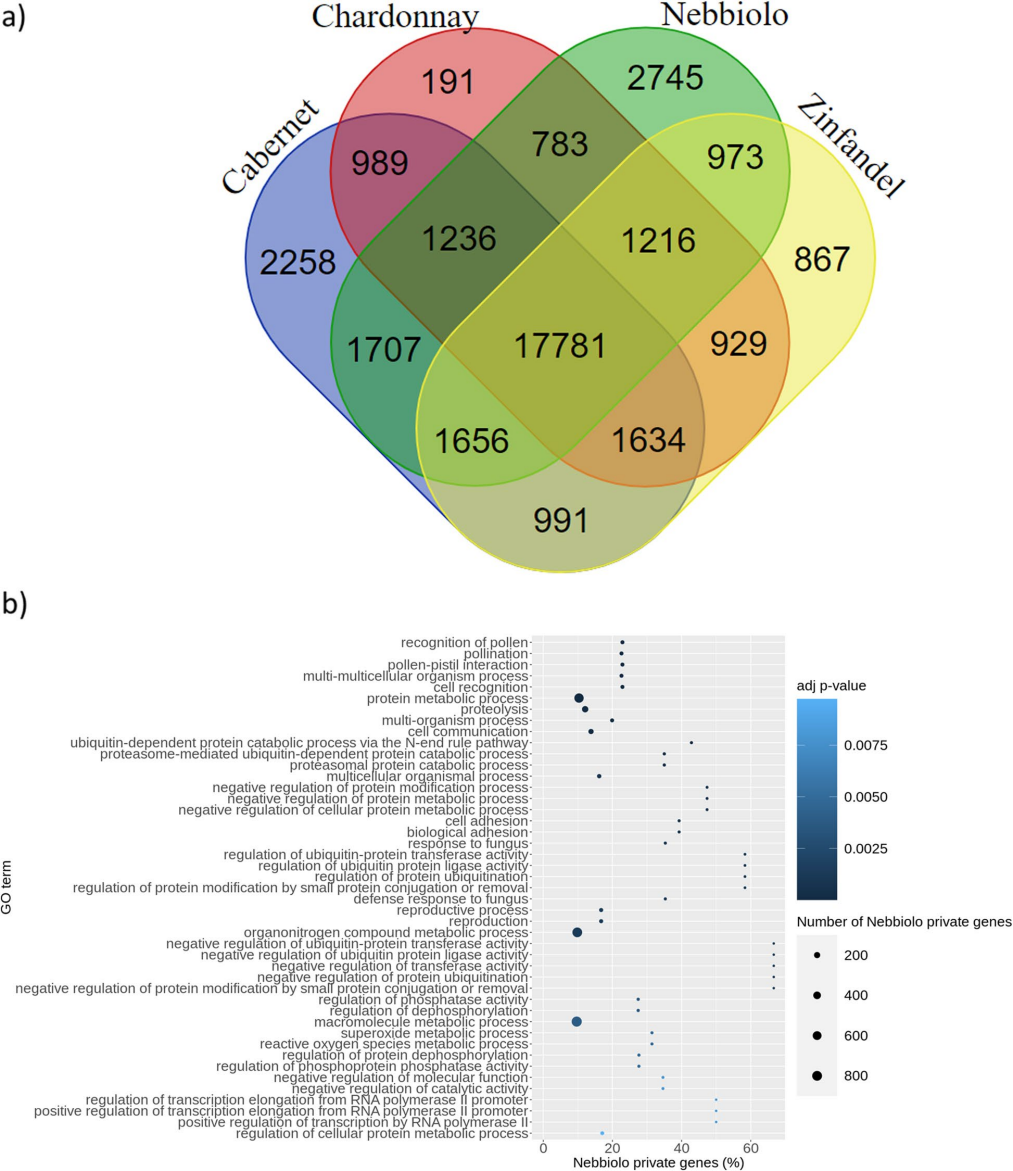
A comparison among ‘Nebbiolo’, ‘Cabernet Sauvignon’, ‘Chardonnay’ and ‘Zinfandel’ gene families. **a**) Venn diagram showing the number of shared and cultivar-specific gene families. **b**) Functional enrichment analysis of the biological processes associated to ‘Nebbiolo’ private genes. For each significantly enriched GO term, the number of ‘Nebbiolo’ associated private genes is represented, along with the percentage of genes associated with a GO that are private to ‘Nebbiolo’. The size and color of the circles represent the number of ‘Nebbiolo’ private genes and the adjusted *p*-value, respectively. Only GO terms with adjusted *p*-value < 0.01 are shown

Annotation was performed separately on the primary assembly and alternative haplotypes. Overall, 50.44% of ‘Nebbiolo’ genome was accounted as repetitive, with Long Terminal Repeats (LTRs) being identified as the most abundant repetitive element class (Table S2). To annotate protein-coding genes, we performed an *ab-initio* prediction supported by carefully filtered hints from publicly available RNA-seq data and proteins from *Arabidopsis thaliana* and *V. vinifera.* A total of 35,038 and 32,865 protein coding genes were identified in ‘Nebbiolo’ primary assembly and alternative haplotypes, respectively (Table 1). The annotated protein coding genes of the primary assembly and alternative haplotypes contained 94.7% and 78.7% of BUSCO genes, respectively. Overall, for both subgenomes combined we could assign a biological function to 87.0% (59,086) of the predicted genes, while a gene ontology (GO) term was assigned to 62.3% (42,271) of them. We performed a comparative analysis by clustering the proteomes of ‘Nebbiolo’, ‘Chardonnay’, ‘Cabernet Sauvignon’ and ‘Zinfandel’, and identified 35,956 gene families containing 202,018 protein coding genes (Fig. 1a). From all the predicted gene families, 17,781 were shared among the four cultivars, and 2,745 gene families were specific for ‘Nebbiolo’. ‘Nebbiolo’ proprietary gene families contained 10,747 protein coding genes, and were significantly enriched for 60 GO biological processes, involving protein and macromolecule metabolic processes, organonitrogen compound metabolic process, phosphorylation and proteolysis (Fig. 1b and Table S3).

### Heterozygous structural variations are abundant between haplotypes

In order to identify heterozygous polymorphisms across ‘Nebbiolo’ CVT 71 genome, we employed three different methodological approaches: PacBio SMRT long-reads (hereafter: SMRT), 10 × Genomics linked-reads (hereafter: 10xG) and genome to genome alignment (hereafter: Genome). SMRT long-reads and 10xG linked-reads generated for clone CVT 71 (Table 2) were aligned to the ‘Nebbiolo’ primary assembly. On the other hand, for the Genome approach, ‘Nebbiolo’ alternative haplotypes were aligned to the ‘Nebbiolo’ primary assembly. The three employed bioinformatic strategies to call heterozygous SVs were further confirmed through PCR experiments. Overall, 70% of the PCRs produced the two expected amplicons, while 20% produced one of the two expected amplicons, while 10% of the reactions did not work (Figure S1 and Table S4).

**Table 2:** Raw genomic data obtained for ‘Nebbiolo’ clones CVT 71 and CVT 185

‘Nebbiolo’ clone	Sequencing platform	Data type	N50 reads/ molecules (bp)	Number of reads/molecules generated	Number of bases generated (Gbp)
CVT 71	PacBio SMRT	Long-reads	27,197	3,286,690	51
CVT 71	Bionano Genomics	Single-molecule maps	241,361	654,883	157
CVT 71	10 × Genomics	Linked-reads	2 × 150	273,179,528	82
CVT 185	10 × Genomics	Linked-reads	2 × 150	261,527,174	78

**Fig. 2:**
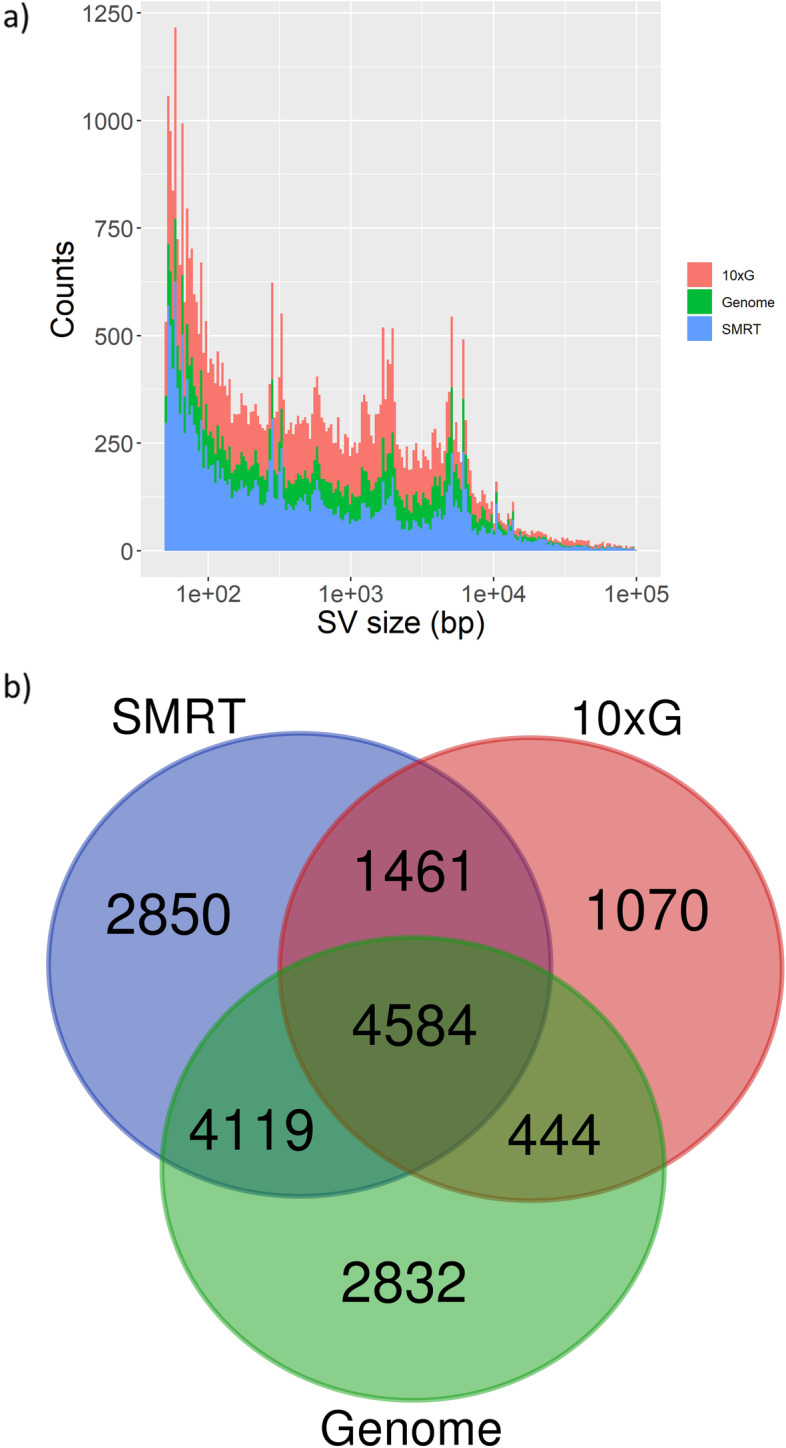
Structural Variants (SVs) between ‘Nebbiolo’ CVT 71 haplotypes identified by three different methodological approaches. **a)** Bar plot showing SVs size distribution. For each size, a coloured bar represents the number of SVs identified by each of the three approaches. The sizes of SVs are represented in log-scale. **b)** Venn diagram showing SVs intersection identified by each approach. Intersection is based on the genomic coordinates at which SVs were called. Abbreviations and colour code for both images: 10xG = 10 × Genomics linked-reads (red); SMRT = PacBio SMRT long-reads (blue); Genome = Genome to genome alignment (green)

Mean coverage reached with each set of genomic data was 59X (SMRT) and 63X (10xG). We observed that the sensitivity of each approach at detecting SVs was quite different. The SMRT approach identified the highest number of SVs (21,241), followed by the Genome (20,010) and 10xG (10,083) approaches (Table 3). In order to use these results as an approximation to compare the relative performance of each approach, SVs were merged both intra and inter-approaches by the adopted software (see Methods section for details) (Fig. 2).

**Table 3:** Structural Variant (SV) types identified comparing ‘Nebbiolo’ CVT 71 haplotypes

SV calling approach	DELs	INSs/DUPs	INVs	TOT	Cumulative size of SVs (Mbp)
PacBio SMRT	13,138	8,016	87	21,241	79.6
10 × Genomics	10,056	18	9	10,083	54.1
Genome	9,845	9,352	87	20,010	52.0

Cumulative size of the non-redundant set of 17,360 merged SVs, identified by considering the three approaches (Fig. 2), represents 17.8% (99.7 Mbp) of the primary assembly, indicating that heterozygous SVs are abundant when comparing ‘Nebbiolo’ haplotypes. In particular, deletions/insertions (> 50 nt) appeared as the most abundant type of SV, suggesting that a large fraction of ‘Nebbiolo’ genome is unbalanced (Table 3). In this direction, we found that 6,534 out of 17,361 SVs (37.6%) overlapped to a gene, indicating high levels of hemizygosity (Table S5). More precisely, SVs affected the CDS regions of 9,367 (26.7%) putative protein coding genes of the primary assembly. Analysis of GO terms showed that many genes associated with 36 different biological processes are affected by heterozygous SVs, including the triterpenoid and beta-glucan biosynthesis (Figure S2 and Table S6).

In regard to the relative performance of the three methodological approaches employed, we observed that from all the SVs identified with SMRT, 45.9% overlapped to SVs identified by 10xG and 66.9% overlapped to SVs identified by the Genome approach. At the same time, 81.8% of the SVs identified by 10xG overlapped to SVs identified by SMRT approach. Finally, 72.7% of the SVs identified with the Genome approach overlapped to SVs identified by SMRT approach (Fig. 2b). Manual inspection of a subset of SMRT and 10xG reads alignments confirmed SVs called using SMRT approach as true variants. Moreover, we observed that many SVs identified with SMRT data were also supported by 10xG reads, despite they were not pinpointed by the variant caller for 10xG data (e.g. Figure S3).

### No evidence of genomic structural variations between two ‘Nebbiolo’ clones

10 × Genomics linked-reads were obtained for clones CVT 71 and CVT 185, representing the ‘Nebbiolo’ biotypes “Michet” and “Lampia”, respectively. Mean molecule lengths of barcoded libraries were 85,986 bp (CVT 71) and 69,603 bp (CVT 185); mean coverage values were 63X (CVT 71) and 60X (CVT 185). Linked-reads were aligned to the ‘Nebbiolo’ CVT 71 primary assembly, to identify putative structural polymorphisms differentiating the two clones. After performing SVs calling and quality filtering, a total of 10,234 SVs were identified for clone CVT 185 and 10,083 SVs for CVT 71, in both cases the great majority were deletions (Table 4).

**Table 4:** Structural Variant (SV) types identified with 10 × Genomics data for ‘Nebbiolo’ clones CVT 71 and CVT 185

SV calling approach	Clone	DELs	INSs/DUPs	INVs	TOT	Cumulative size of SVs (Mbp)
10 × Genomics	CVT185	10,215	11	8	10,234	51.8
10 × Genomics	CVT71	10,056	18	9	10,083	54.1

The intersection of the sets of SVs called for CVT 71 and CVT 185 (10xG) suggested that 1,244 SVs occurred only in clone CVT 185 and not in CVT 71 (Figure S4). However, after manual inspection, none of the 1,244 variants were confirmed as occurring only in clone CVT 185, because similar number of reads supported that SV for both clones (e.g. Figure S5), suggesting a high number of false negatives in the 10xG-based SVs call set.

### Structural variations between cultivars are more frequent than between haplotypes

**Fig. 3:**
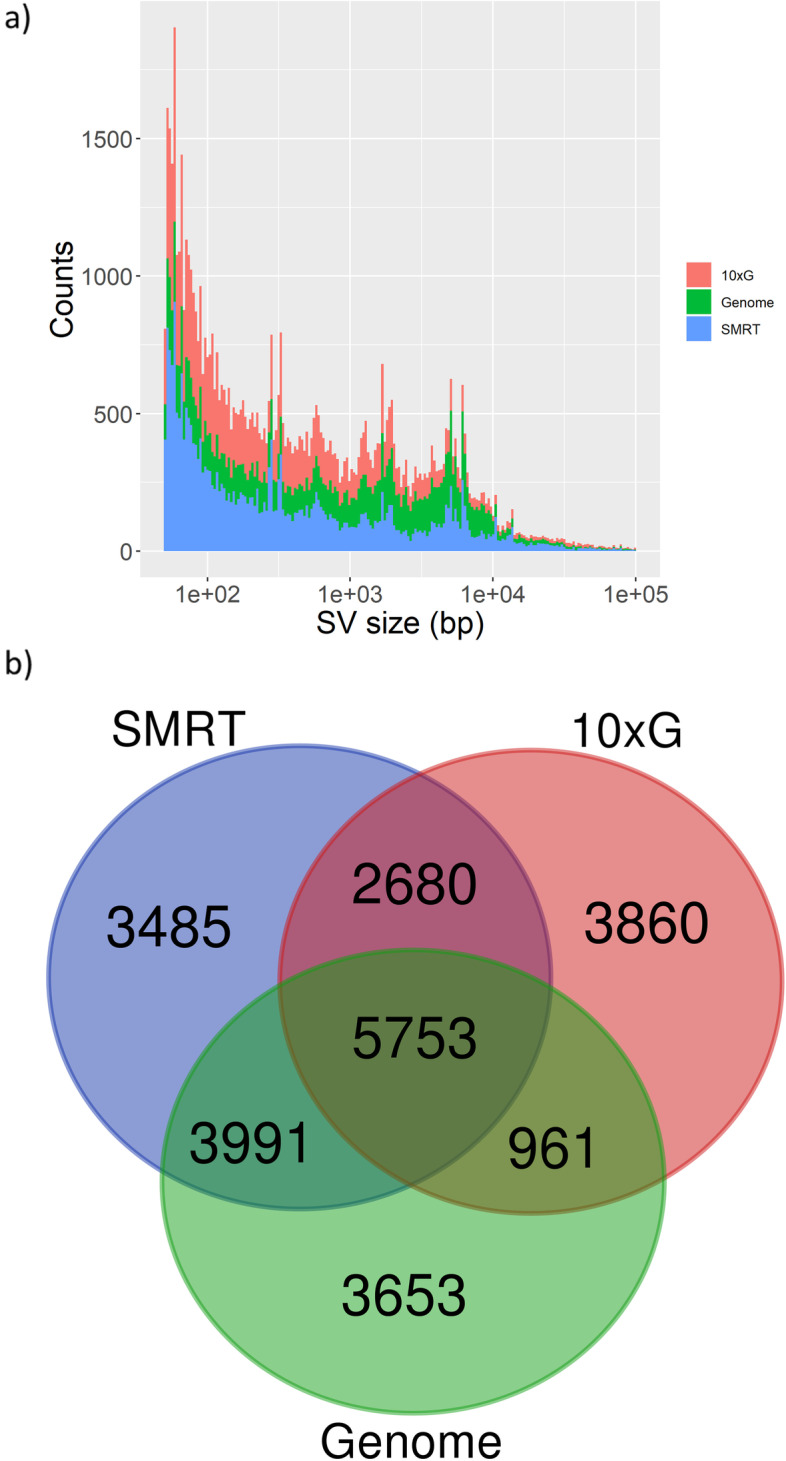
Structural Variants (SVs) between ‘Nebbiolo’ and ‘Cabernet Sauvignon’ identified with three different methodological approaches. **a**) Bar plot showing the size distribution of the called SVs. For each size, a coloured bar represents the number of SVs identified by each approach. The sizes of SVs are represented in log-scale. **b**) Venn diagram showing SVs intersections, based on the genomic coordinates at which SVs were called. Abbreviations and colour code for both images: 10xG = 10 × Genomics (red); SMRT = PacBio SMRT (blue); Genome = Genome to genome alignment (green)

We investigated the occurrence of SVs between grapevine cultivars, by comparing ‘Nebbiolo’ to ‘Cabernet Sauvignon’ (hereafter: Cabernet). On one side, we aligned ‘Nebbiolo’ genomic data to a Cabernet (clone: FPS 08) primary assembly publicly available [24], the mean coverage values were 39X (SMRT) and 57X (10xG). On the other side, we aligned ‘Nebbiolo’ primary assembly to ‘Cabernet’ primary assembly, using the Genome approach. After performing the variant calling, once again the method based on SMRT data identified the highest number of SVs (27,319), followed by the Genome approach (24,996) and 10xG (19,016) approaches (Table 5). SVs were merged both intra and inter approaches by the adopted software, to compare the performance of the different approaches (see Methods) (Fig. 3).

**Table 5:** Structural Variant (SV) types identified comparing ‘Nebbiolo’ to ‘Cabernet Sauvignon’

SV calling approach	DELs	INSs/DUPs	INVs	TOT	Cumulative size of SVs (Mbp)
SMRT	17,622	9,632	65	27,319	136.1
10xG	18,975	24	17	19,016	141.8
Genome	12,509	10,823	1,664	24,996	76.4

Cumulative size of the non-redundant set of 24,383 merged SVs identified by considering the three approaches (Fig. 3) represents 44.2% (261.4 Mbp) of ‘Cabernet’ primary assembly; this value indicates that SVs between the two cultivars are abundant. In particular, deletions (> 50 nt) appeared as the most abundant type of SV detected by the three approaches (Table 5). In respect to insertions/duplications, we observed that the SMRT and Genome approaches outperformed 10xG at detecting this SV type. Overall, we found that 8,094 out of 24,383 SVs (33.2%) overlapped to a gene (Table S5), affecting 16,109 (43.9%) genes across ‘Cabernet’ genome. However, GO enrichment analysis showed that these genes were not significantly overrepresenting a particular biological process. In regard to the relative performance of the three methodological approaches, we observed that from all the SVs identified by the SMRT approach, 53.0% overlapped to SVs identified by 10xG, and 61.2% overlapped to SVs identified by the Genome approach. At the same time, 63.6% of SVs identified by 10xG overlapped to SVs identified by SMRT. Finally, 67.9% of SVs identified by the Genome approach overlapped to SVs identified by SMRT (Fig. 3b). Manual inspection of these variants confirmed that any given SV called by the SMRT approach is likely to be a true variant, regardless of the other two approaches supporting that same SV or not (e.g. Figure S6), while SVs not identified by SMRT approach seem to have lower confidence.

## Discussion

Significant innovations in genomic platforms have recently enabled a deeper comprehension of plant genomes complexity, allowing the extensive phasing of genome assemblies and SVs direct identification [3, 22]. In particular, a detailed characterization of both haploid complements of the diploid genome of some grapevine cultivars has been recently achieved [2, 23, 24]. Here, we used long-range genomic data to assemble de novo a genome for cultivar ‘Nebbiolo’. This assembly was employed to survey the incidence of SVs across different organizational levels and interpret their functional implications on grapevines genomes, by comparing haplotypes, clones and cultivars. Finally, we discussed the relative performance of the methodological approaches employed here at detecting SVs.

In order to maximize our ability to detect SVs, we assembled de novo and annotated a genome for ‘Nebbiolo’ (clone CVT 71). The size of the obtained primary assembly (561 Mbp) turned 15% bigger than the partially inbred ‘Pinot Noir’ PN40024 (486 Mbp) [29] and ‘Chardonnay’ (490 Mbp) [14] assemblies. At the same time, our assembly resulted smaller than those of other *V. vinifera* cultivars, e.g. ‘Cabernet Sauvignon’ (590 Mbp) [24], ‘Zinfandel’ (591 Mbp) [25], ‘Chardonnay’ (605 Mbp) [2] and ‘Carménère’ (623 Mbp) [23]. Variations in the primary assembly size might be mainly explained in terms of retention of both copies of some heterozygous regions in the primary assembly [23]. In fact, assemblies [2, 24, 25] and [23], which show a bigger size than the expected for grapevines haploid complement, were obtained using the diploid-aware FALCON-Unzip pipeline. On the other hand, only Chardonnay [14] was processed with bioinformatic tools aimed at optimizing reassignment of allelic contigs, based on homology and read-coverage [30]. Supporting this observation, two assemblies for ‘Chardonnay’ that differ in the latter methodological aspect have been reported [2, 14] showing 20% difference in size. Here, primary contigs produced by Falcon-Unzip resulted 37% bigger than the primary assembly, meaning that grapevine’s high heterozygosity makes challenging to correctly discriminate between primary and alternative contigs, leading to imprecise estimates of the haploid genome size. In our case, long-range genomic data provided by optical consensus maps proved to be valuable for scaffolding contigs that came from the same haplotype, notably increasing the N50 value (Table 1 and Table S1). This improved the contiguity and possibly allowed reducing haplotype switch errors, thus smoothing the identification of homologous regions. Starting from such assembly, genome annotation showed that more than half of ‘Nebbiolo’ genome is repetitive, with LTRs identified as the most abundant repetitive element class, as reported for other grapevine cultivars genomes [11, 23]. We also compared ‘Nebbiolo’ to other *V. vinifera* cultivars proteomes, thus identifying cultivar-specific gene families. The functional enrichment analysis of ‘Nebbiolo’ proprietary gene families led to the identification of 60 enriched biological processes GO terms, including response to fungal infections among others (Fig. 1b and Table S3). This result reinforces previous observations obtained from short-reads sequencing [10] and from the comparison between ‘Nebbiolo’ and other cultivars, in response to multiple pathogen infections [31] and environmental factors [32]. ‘Nebbiolo’ has a number of proprietary genes specific for the response to pathogens, as well as mutations associated with common defence genes [10] and a differential regulation of their expression probably linked to specific responses towards particular pathogens [33].

To explore the occurrence and functional implications of SVs in ‘Nebbiolo’ at different organizational levels, we performed comparisons between the two haplotypes of the same individual, between two clones and between two cultivars. Our analyses comparing haplotypes showed that a significant proportion of ‘Nebbiolo’ genome is affected by heterozygous SVs. In particular, the cumulative size of SVs identified by long-reads represents 17.8% of the primary assembly size, a value considerably higher than the 6.94% reported for ‘Zinfandel’ [25] but similar to the 15.1% reported for ‘Chardonnay’ [2]. We are aware that the reported SVs cumulative sizes (between haplotypes and cultivars) might be an upper-bound of the true value, because variants detected only with 10xG were also considered. Nonetheless, the laboratory validation of the different approaches and  thresholds employed here to call heterozygous SVs provide more certainty on the obtained results. The majority of the PCRs (70%) produced the expected amplicons, a small amount (10%) of the reactions did not produce amplicons at all, while 20% percent of the cases produced only one of the two expected amplicons. The latter might have alternative explanations, one of them is that PCR is also a non-error-free method and allelic dropout (among other issues) might produce that kind of results [34]. We observed that many of the called SVs between haplotypes overlapped to coding genes (Table S5). These findings further support the observations that hemizygosity is rampant across grapevine’s genomes [2] and the proposed high diversity between ‘Nebbiolo’ -still unknown- parental cultivars [10], as all extant grapevine cultivars originated from out-crossing two pre-existing cultivars [12]. In regard to clonal comparison, few studies have performed a genome-wide analysis of somatic mutations accumulated in different clones of the same grapevine cultivar [10, 14, 25, 35]. Among the latter works, only Vondras et al. [25] studied the occurrence of SVs among 15 ‘Zinfandel’ clones, based solely on short-reads data, and they observed that SVs are less frequent than SNVs. Even though thousands of SVs were reported among ‘Zinfandel’ clones, authors pointed out that additional work should be undertaken to confirm these variants [25]. Results obtained here add further evidence on the two following concepts: firstly, a thorough validation is essential for spotting spurious SV calls [13, 27, 36]. Here, none of the spotted SVs that were supposed to occur in one clone and not in the other passed the manual inspection criteria. Secondly, as observed for ‘Zinfandel’, SVs differentiating ‘Nebbiolo’ clones are much less frequent than SNVs [10]. Even though clones CVT 71 and CVT 185 represent two different biotypes, with phenotypic and genetic (SNVs) differences [10], here we were not able to retrieve a single SV differentiating them. On the other hand, when cultivars ‘Nebbiolo’ and ‘Cabernet Sauvignon’ were compared, the total number of SVs was higher than that observed between haplotypes and similar to that observed when ‘Chardonnay’ and ‘Cabernet Sauvignon’ were compared [2]. The functional enrichment analysis of genes affected by SVs between ‘Nebbiolo’ haplotypes identified that genes involved in the triterpenoid biosynthesis and metabolic process were particularly affected by heterozygous SVs (Fig. S2). Triterpenoids are lipids that comprise the main compound of the cuticular wax coating the berries [37]. These lipids serve as protection against biotic (pathogen defence) and abiotic (preventing desiccation) stresses; they also have technological importance for the wine industry because of their nutraceutical properties [37]. On the other hand, no GO term was particularly enriched when ‘Nebbiolo’ and ‘Cabernet Sauvignon’ were compared, possibly because the high number of genes affected by SVs saturated the enrichment analysis (see Methods).

We used the data obtained from comparisons between haplotypes and between cultivars as an approximation to evaluate the relative performance of the employed approaches at detecting SVs. SMRT long-reads turned to be the approach identifying the highest number of reliable SVs, followed by the Genome alignment and by 10xG linked-reads. Considering SVs called by SMRT long-reads as our ‘gold standard’ [2] linked-reads proved to be almost as precise although not as sensitive, while the genome alignment approach proved to be both precise and sensitive. Genome alignment has the advantage that it doesn’t require access to raw reads, indirectly incorporating evidence from all data sources used for the assembly process; therefore, its performance strongly depends on the quality of the assembly. Deletions (DELs) were the most abundant type of SVs detected, while insertions (INSs) were detected in lower number. This is similar to that observed for short-reads and could be explained by the higher algorithmic difficulty of calling insertions through mapping approaches [18] and from the biased introduced by 10xG SV caller (Long Ranger), which detects duplications but not insertions [38]. Overall, the difference in the number of SVs detected by the three approaches and the low number of SVs detected simultaneously by all of them (Fig. 2 and 3) may be explained by multiple factors. First, reads/fragments length have a strong impact on the portion of the assembly which can be genotyped, and 10xG linked-reads only partially overcome issues typical of short-reads [39, 40]. Second, different approaches may show performances strongly dependent on the specific class of SVs, but combining multiple predictions to obtain a more reliable set of variants is still an active research area [17, 20]. In this context, our results highlight the importance of performing a thorough manual validation of the SVs, in order to have more certainty of the called variants. Finally, we are confident that the assembly reported here along with other genome assemblies produced for different cultivars [2, 14, 23–25] will propel the construction of a pan-genomic infrastructure for grapevines [41]. In combination with graph-based toolkits for their visualization and analysis, such infrastructures should increase the accuracy of the SVs calling process [42, 43].

## Conclusions

We found that SVs accumulate at different rates in grapevines, depending on the organizational level under study. The obtained results indicate that SVs differentiating clones of the same cultivar are infrequent, if not totally absent. This is contrary to the observed between haplotypes of the same individual and between cultivars, where SVs are abundant and accumulate at higher rates. In particular, we added further evidence on the unbalance condition that characterizes grapevine genomes, affecting a great number of genes involved in relevant functional processes. Finally, after evaluating different approaches to survey the occurrence of SVs, we observed that SMRT long-reads is the most suited method for this aim. Future studies should further investigate the transcriptomic and phenotypic consequences of the high hemizygosity affecting genes involved in relevant biological processes in ‘Nebbiolo’.

## Methods

### Samples’ origin and genomic data generation

Samples from clones CVT 71 and CVT 185 represent the ‘Nebbiolo’ biotypes “Michet” and “Lampia”, respectively. The material was obtained from registered accessions at the Institute for Sustainable Plant Protection, National Research Council (Turin, Italy). Samples employed to assemble the *V. vinifera* L. cv. Nebbiolo reference genome correspond to clone CVT 71. For PacBio sequencing, DNA was extracted at the Functional Genomics Laboratory (University of Verona, Italy), from 1 g of young leaves. We used the cetyltrimethylammonium ammonium bromide (CTAB) extraction buffer [44] modified from [45] and [46] as described in [24], combined with PacBio Guidelines for gDNA clean-up. The purity of extracted DNA was assessed using NanoDrop™ 1000 Spectrophotometer (Thermo Scientific, Germany). Genomic DNA concentration was fluorometrically measured combining dsDNA Broad Range Assay Kit with Qubit® 4.0 (Thermo Fisher Scientific, Waltham, USA); the size of DNA fragments was evaluated using the CHEF Mapper electrophoresis system (Bio-Rad Laboratories, California). Genomic DNA (16 μg) was used to prepare a single‐molecule real‐time (SMRT) bell library according to the manufacturer's protocol (Pacific Biosciences; 30-kb template preparation using BluePippin (SageScience) size selection system with a 20-kb cut-off). Sequencing was performed on a PacBio RS II platform (Pacific Biosciences, CA, USA) producing 3,286,690 reads with a N50 of 27,179 bp and a total of 51 Gbp of SMRT data using PacBio P6‐C4 chemistry. Library preparation and sequencing were performed at the University of California Davis (California, USA).

Bionano Genomics mapping is based on the enzymatic digestion of high-molecular weight DNA molecules by a nicking enzyme, followed by the incorporation in the nicks of a fluorescent nucleotide. Labelled molecules are scanned and distances between labels are recorded after image digitalization [47]. Here, we used young grapevine plants of clone CVT 71 maintained under in vitro conditions on solid sterile culture media. High Molecular Weight DNA was extracted from 1 g of freshly harvested leaves using the IrysPrep Plant Tissue DNA Isolation Protocol (Bionano), with minor adjustments as described in [26]. The size of the extracted DNA was verified by Pulsed-Field-Electrophoresis (PFGE). DNA (510 ng) was labelled and stained using 3.4 µl of Nb.BssSI (20 U/µl) nicking endonuclease in combination with the NLRS DNA labelling kit (Bionano Genomics). Finally, the labelled DNA was loaded on an Irys chip. DNA extraction, labelling and image acquisition were performed at the Functional Genomics Laboratory (University of Verona, Italy).

For 10 × Genomics library preparation of clones CVT 185 and CVT 71, high-molecular-weight DNA was extracted from a nuclear preparation obtained from 1 g of young leaves. Tissue grounded in liquid nitrogen was resuspended in NIBTM (10 mM Tris pH 8, 10 mM EDTA pH 8, 0.5 M Sucrose, 80 mM KCl, 8% (w/v) PVP-10, 100 mM Spermine, 100 mM Spermidine, pH 9.0) supplemented with 0.5% Triton-100 and 0.2% beta-mercaptohetanol and kept on ice for 30 min. The tissue homogenate was filtered first through a 100 µm and then through a 40 µm cell strainer, then centrifuged at 2500 g for 20 min at 4 °C in a swing bucket rotor. Nuclei pellet was resuspended gently and washed with 30 ml of cold buffer and spun at 60 g for 2 min at 4 °C with no deceleration to remove tissues debris. The supernatant containing nuclei was filtered through at 40-µm cell strainer and spun to pellet the nuclei again at 2500 g for 20 min. The latter step was repeated until a white nuclei pellet and a clear supernatant were obtained. DNA was extracted from the isolated nuclei pellets using the Qiagen Genomic tip-100 (Qiagen) following the manufacturer’s instructions. Size of extracted DNA was verified by Pulsed-Field-Electrophoresis (PFGE). A 10 × GEM library was constructed according to manufacturer’s recommendations (10 × Genomics) starting from 10 ng HMW DNA. DNA extractions and library preparations were performed at the Functional Genomics Laboratory (University of Verona, Italy). Libraries were quantified by qPCR and sequenced at Macrogen Inc. (Seoul, South Korea), using an Illumina HiSeq X Ten instrument.

### De novo Genome assembly and scaffolding of the ‘Nebbiolo’ genome

A preliminary de novo assembly based on PacBio SMRT long-reads from CVT 71 clone was performed using FALCONUnzip-DClab [23]. This is a pipeline based on FALCON Unzip v1.7.7 [24] and Damasker v1.0p1 [48] available at https://github.com/andreaminio/FalconUnzip-DClab. In detail, repeats were first masked using TANmask and REPmask modules in Damasker. Reads were corrected with Falcon Correct and repeats were masked also on corrected reads; afterwards, reads were assembled using FALCON. Multiple parameters were tested to produce the least fragmented assembly, and haplotype reconstruction of the best assembly was performed with FALCON Unzip. Polishing of the preliminary assembly was performed using Arrow algorithm from GenomicConsensus v2.3.3. package (Pacific Biosciences). Illumina data for clone CVT 71 produced elsewhere [10] was used to polish the assembly. Illumina reads were mapped to the preliminary assembly with BWA mem v0.7.17 [49] and the resulting *bam* file was used for polishing with Pilon v1.23 [50] in diploid mode ($PILON –genome $DRAFT –frags $BAM –output $SAMPLE_NAME"_pilon_x"$ROUND_NUM –diploid –outdir $WORKING_DIR –vcf –changes –threads $THREADS –Xmx250G). 10 × Genomics data for clone CVT 71 was  used to attempt a first round of scaffolding with ARCS v1.1.1 [51] but this provided very marginal improvements in assembly N50, and this step was therefore skipped. Bionano optical maps were employed to correct mis-assemblies by anchoring NGS contigs to consensus maps with Bionano Solve v3.4 hybrid scaffolding pipeline. This was performed with the following settings: expected genome size of 0.5 Gbp, no preassembly, cut CMPR, non-haplotype, extend and split, cut segdups and Irys instrument. In particular, single-molecule maps were assembled into consensus maps, that were used to produce a hybrid assembly. To reduce redundancy and separate the primary assembly from the alternative haplotypes, two rounds of purging were performed with purge_haplotigs v1.1.0 [30]. This was performed by identifying shorter homologous sequences, based on coverage depth and alignment identity, and placing them into a separate file. Moreover, sequences with average coverage lower than 15X or greater than 115X were categorized as assembly artefacts and were removed from the assembly. Illumina reads were mapped to the primary assembly and to the alternative haplotypes separately with BWA mem, and two additional rounds of polishing using Pilon were performed. To assess the gene content, a BUSCO v4.1.2 [52] search was applied to the primary assembly and alternative haplotypes using the *eudicotyledons_odb10* database. The obtained primary assembly (560 Mbp) was used as the ‘Nebbiolo’ reference for SVs calling. Assembly statistics were calculated with assembly-stats v1.0.1 (https://github.com/sanger-pathogens/assembly-stats).

### ‘Nebbiolo’ genome annotation

Genome annotation was performed separately for the primary assembly and for the alternative haplotypes, hereafter the term ‘assembly’ will be used to refer to both of them. First, repetitive elements were identified using RepeatModeler v2.0.1 [53] with LTR structural search pipeline. Repetitive elements were then used for soft-masking the genome assembly using RepeatMasker v4.1.1 [54]. Publicly available RNA-seq datasets [10, 55] (PRJNA477842 and PRJNA387534) were aligned to the assembly with HISAT2 v2.2.1 [56] using the option *max_intron_length* = *60kbp*. Proteins of *Arabidopsis thaliana* TAIR10 [57] and *V. vinifera* L. cv. Pinot Noir [1] obtained from Phytozome v13 [58] were aligned to the assembly with GenomeThreader v1.7.1 [59]. BUSCO v4.1.2 [52] was then used to train a model using eudicots BUSCO genes. Intron hints derived from RNA-seq data were retained if they were confirmed by at least 10 reads spanning across the junction and were provided to the predictor software. Finally, structural genome annotation was performed with AUGUSTUS v3.3.3 [60] using the trained model, with proteins and RNA-seq alignments used as hints. The function of the annotated protein-coding genes was identified using a custom script which integrates homology, orthology information and identification of functional domains. In brief, predicted proteins were aligned to TAIR10 annotation with Blast v2.2.28 + [61] and the top hit was used to infer a function. Predicted proteins were also compared to orthologous proteins annotated in Arabidopsis TAIR10 [57] and in other grapevine genomes: PN40024 [1], ‘Cabernet Sauvignon’ [11] and ‘Chardonnay’ [2]. In addition, protein domains and motifs were searched with InterProScan v5.46–81.0 [62] with default databases. Orthofinder v2.4.0 [63] was used to define the number of shared gene families among ‘Nebbiolo’, ‘Cabernet Sauvignon’, ‘Chardonnay’ and ‘Zinfandel’ based on their proteomes, and the output was represented using Venn diagrams drawn with the web tool http://bioinformatics.psb.ugent.be/webtools/Venn/. Gene Ontology enrichment analysis of genes belonging to private gene families was performed with BiNGO [64], employing FDR-correction and a *p*-value threshold of 0.05. This output was visualized with R Bioconductor package ggplot2 [65].

### Structural variants identification

For comparison between haplotypes of the same individual, the obtained ‘Nebbiolo’ primary assembly was used as the reference genome. PacBio SMRT long-reads were aligned to the reference using NGMLR v0.2.7 [36]. SVs were called using Sniffles v0.1.12 [36] with default parameters and we removed all SVs with the IMPRECISE and non-PASS flags. 10 × Genomics linked-reads for clone CVT 71 were aligned to the reference genome and SVs were called using Long Ranger v2.2.2 with default parameters, variants with non-PASS flag were discarded. For the genome alignment approach, nucmer and delta-filter from the MUMmer4 package [66] were used to align the ‘Nebbiolo’ alternative haplotypes to the ‘Nebbiolo’ primary assembly (nucmer -maxmatch -noextend), and retain one-to-one alignments with a minimum alignment length of 1,000 bp (delta-filter -1 -l 1000). NucDiff [67] was then used to extract the features and coordinates of SVs. The obtained *gff* files by NucDiff were converted to *bed* files after removing all SVs that could not be classified as deletions, insertions, duplications or inversions, and the *bed* file was converted to *vcf* format with SURVIVOR bedtovcf 1.0.7 [68]. SVs identified with the three methods that overlapped to regions containing ambiguous nucleotides were removed with Bedtools intersect v2.28.0 [69]. Retained SVs were merged using SURVIVOR merge v1.0.7 [68] setting 10 kbp as the maximum allowed distance between starting and ending breakpoints of different SVs to be considered as the same one. In particular, the merging is based on a two-step process: i) in the intra-approach merging step, proximate SVs identified with each approach are merged independently to better cope with noisy alignments; ii) in the inter-approach merging step, intra-approach merged SVs identified with different approaches are merged together. Translocations were excluded from the analysis, because they turned technically difficult to process with the implemented bioinformatic pipelines. The main issue is that each variant caller represents translocations in different ways: either as a pair of breakpoints with SVTYPE = BND (Sniffles and Longranger) or as a single event with Name = translocation-overlap or Name = translocation-insertion (NucDiff). We also removed all SVs < 50 bp from the variants list, because these are defined as InDels rather than SVs [20]. For comparisons between clones (CVT 71 vs. CVT 185), the obtained ‘Nebbiolo’ primary assembly was used as the reference genome, and SV calling was performed with 10 × Genomics linked-reads for the two clones as previously described. Finally, for comparisons between cultivars with the genome alignment approach, ‘Cabernet Sauvignon’ primary assembly [24] was used as the reference genome. SV calling was performed as previously described, but here the ‘Nebbiolo’ primary assembly was aligned to the ‘Cabernet Sauvignon’ primary assembly. The sizes of the final set of SVs identified by the three approaches were plotted in R v3.6.0 with ggplot2 package [65]. The cumulative size of SVs was calculated adding the absolute sizes of the retained SVs.

Manual inspection for SVs validation was performed with IGV genome browser v2.4.17, and the criteria to select the inspected SVs was based on the obtained Venn diagrams. More precisely, we randomly chose three SVs for each of the subsets identified by each platform, both at the individual and cultivar levels, totalling 42 SVs. At the same time, with the aim to identify clone specific variants, all 1,244 SVs called only for clone CVT 185 were manually inspected using Samplot v1.0.20 [70]. SVs were considered as validated if they were supported by at least four non-reference reads (lower threshold). PCR experiments were conducted to further corroborate the three described approaches to call heterozygous SVs between CVT 71 haplotypes, as well as the chosen lower threshold to consider a variant as true. We chose five SVs for each of the three approaches and five SVs supported by the lower threshold. In total 20 SVs were corroborated through PCR experiments; all chosen SVs were heterozygous deletions. For each PCR two amplicons were expected, one longer amplicon without the SV (reference allele) and one shorter amplicon containing the deletion (non-reference allele). See Supplementary Methods and Table S4 for more details on the PCR experiments. Finally, Gene Ontology enrichment analysis of genes affected by heterozygous SVs in ‘Nebbiolo’ and ‘Cabernet Sauvignon’ genome was performed with BiNGO [64] setting FDR-correction and a p-value threshold of 0.05.

## Supplementary Information

**Additional file 1.**

**Additional file 2.**

## Data Availability

The *V. vinifera* L. cv. Nebbiolo raw sequencing reads have been deposited in the SRA (Sequence Read Archive) data resource of the NCBI with the Bioproject ID PRJNA746794. The genome assembly files of primary assembly and alternative haplotypes with their gene and repeat annotations are available at figshare https://doi.org/10.6084/m9.figshare.15023097.
