# Supplementary material for: ‘Nebbiolo’ genome assembly allows surveying the occurrence and functional implications of genomic structural variations in grapevines (Vitis vinifera L.)
Source: BMC Genomics. 2022 Feb 24;23:159. doi: 10.1186/s12864-022-08389-9 (PMC8867635; doi:10.1186/s12864-022-08389-9)
Supplement: Supplementary file 1 — Additional file 1. [file 12864_2022_8389_MOESM1_ESM.docx]

**‘Nebbiolo’ genome assembly allows surveying the occurrence and functional implications of genomic structural variations in grapevines (*Vitis vinifera* L.)**

**Authors**

Simone Maestri, Giorgio Gambino, Andrea Minio, Irene Perrone, Emanuela Cosentino, Barbara Giovannone, Giulia Lopatriello, Luca Marcolungo, Massimiliano Alfano, Stephane Rombauts, Dario Cantu, Marzia Rossato, Massimo Delledonne and Luciano Calderón

**Supplementary Figures**

**
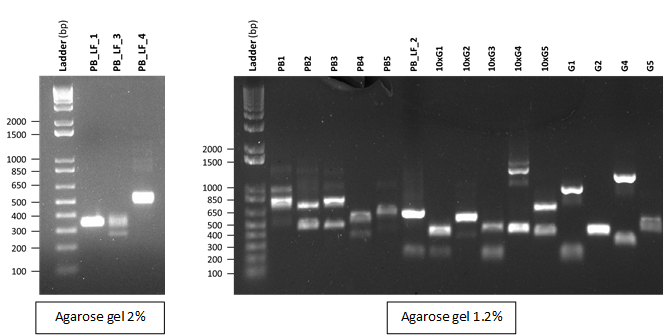
**

**Figure S1. PCR experiments for heteorzygous SVs (deletions) validation. Electrophoresis gel showing the obtained amplicons for the 18 properly working pair of primers. See Table S4 for more details on abreviations and expected results for each experiment.**

**
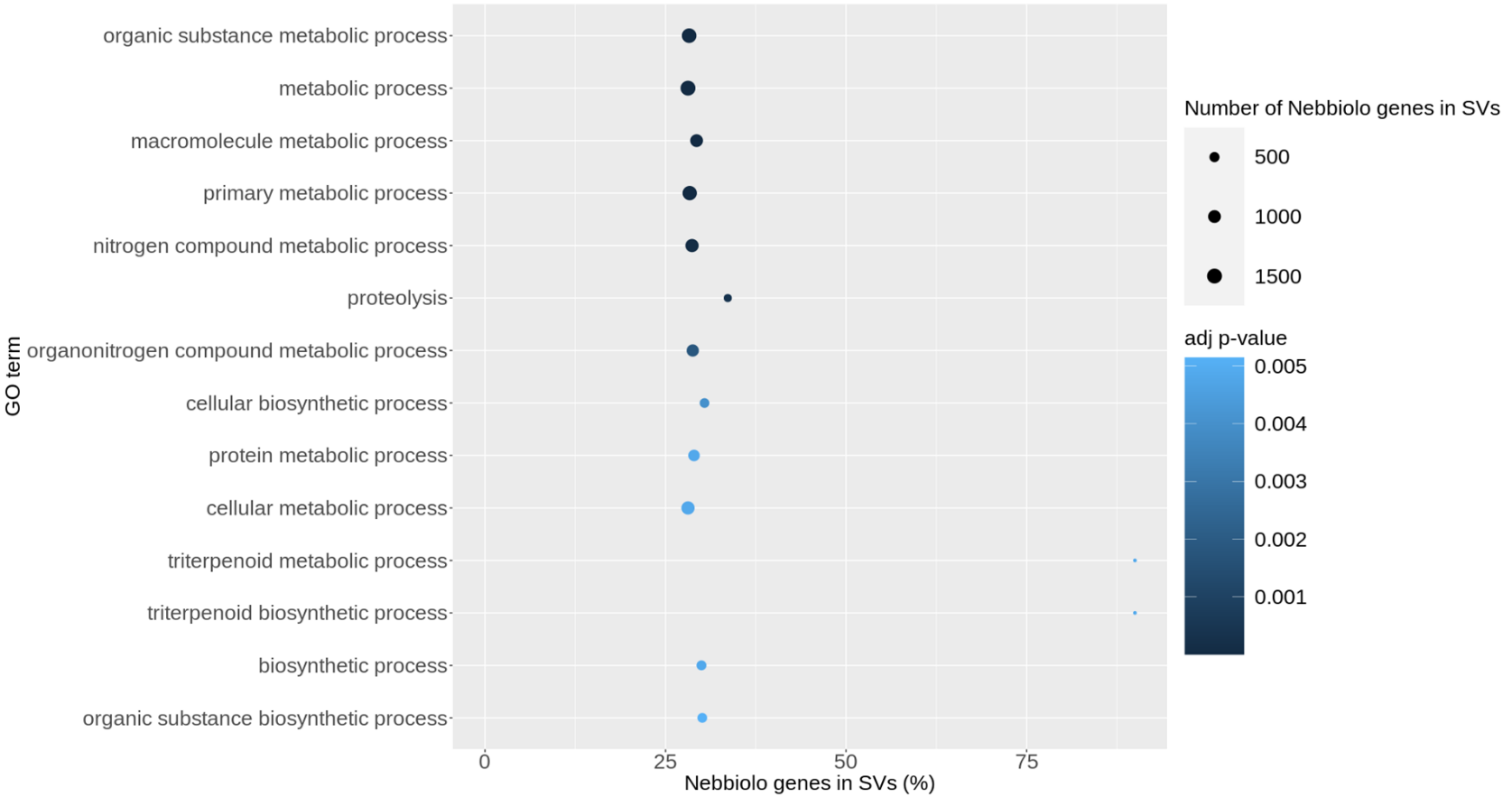
**

**Figure S2. Functional enrichment analysis of biological process for genes located in SVs occurring between ‘Nebbiolo’ haplotypes.** For each significantly enriched GO term, the number of ‘Nebbiolo’ genes in SVs is represented, along with the percentage of genes associated with a GO that are affected by SVs. The size and color of points represent the number of ‘Nebbiolo’ genes in SVs and the adjusted p-value, respectively. Only GO terms with adjusted p-value < 0.01 are shown.

**
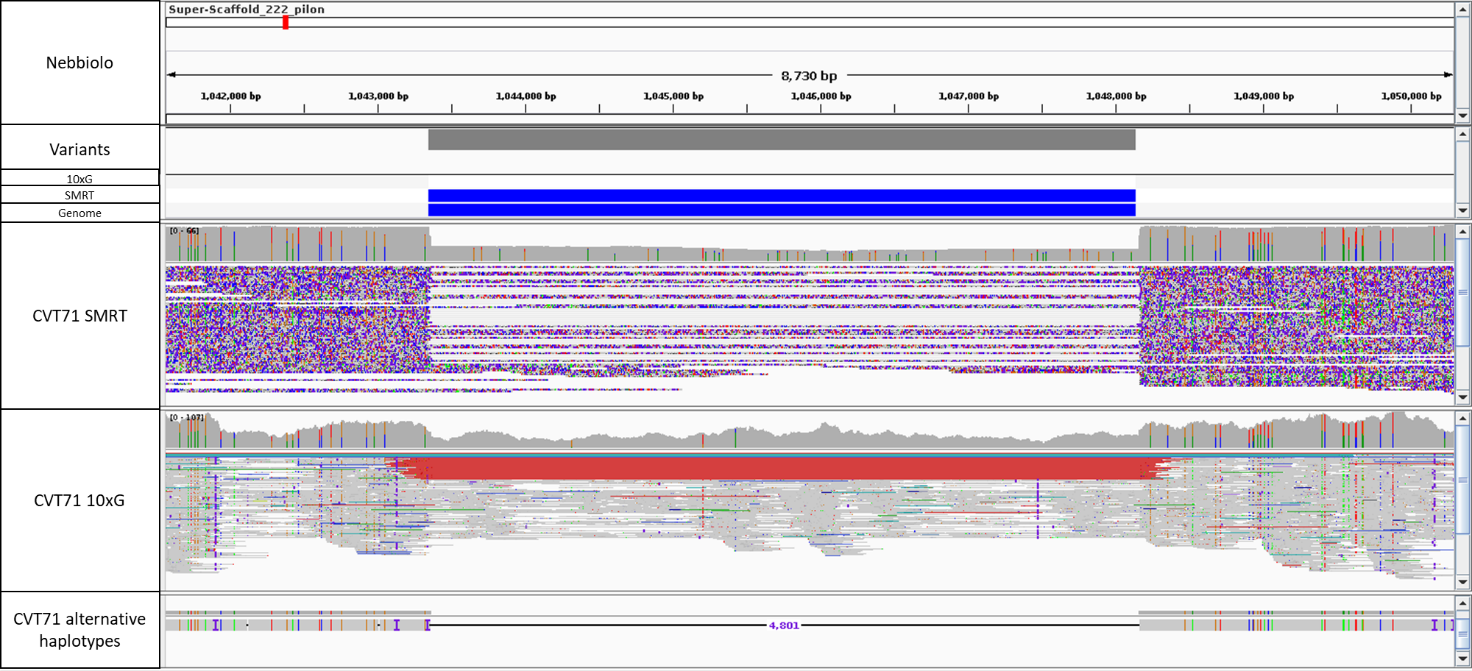
Figure S3. IGV screenshot of a validated heterozygous SV in ‘Nebbiolo’ genome.**

**
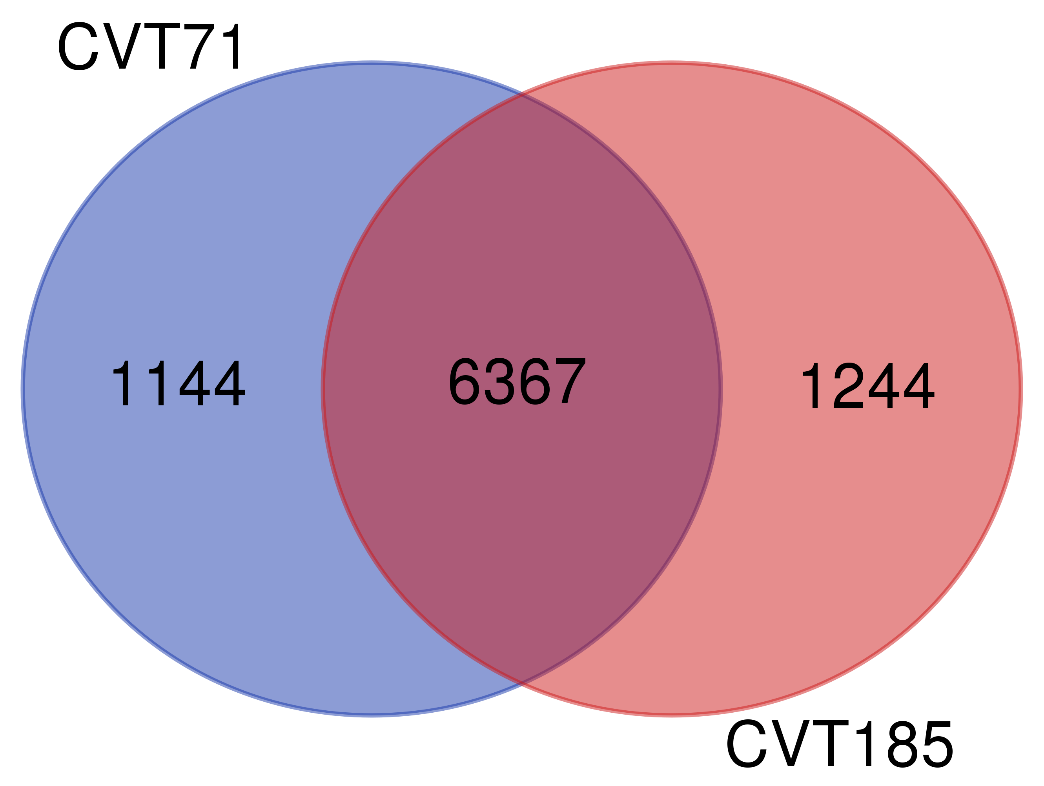
**

**Figure S4. Intersection of Structural Variants (SVs) called for two ‘Nebbiolo’ clones.** The bioinformatic analysis suggests the presence of 1,244 SVs occurring in CVT 185 clone, that are not shared with clone CVT 71.


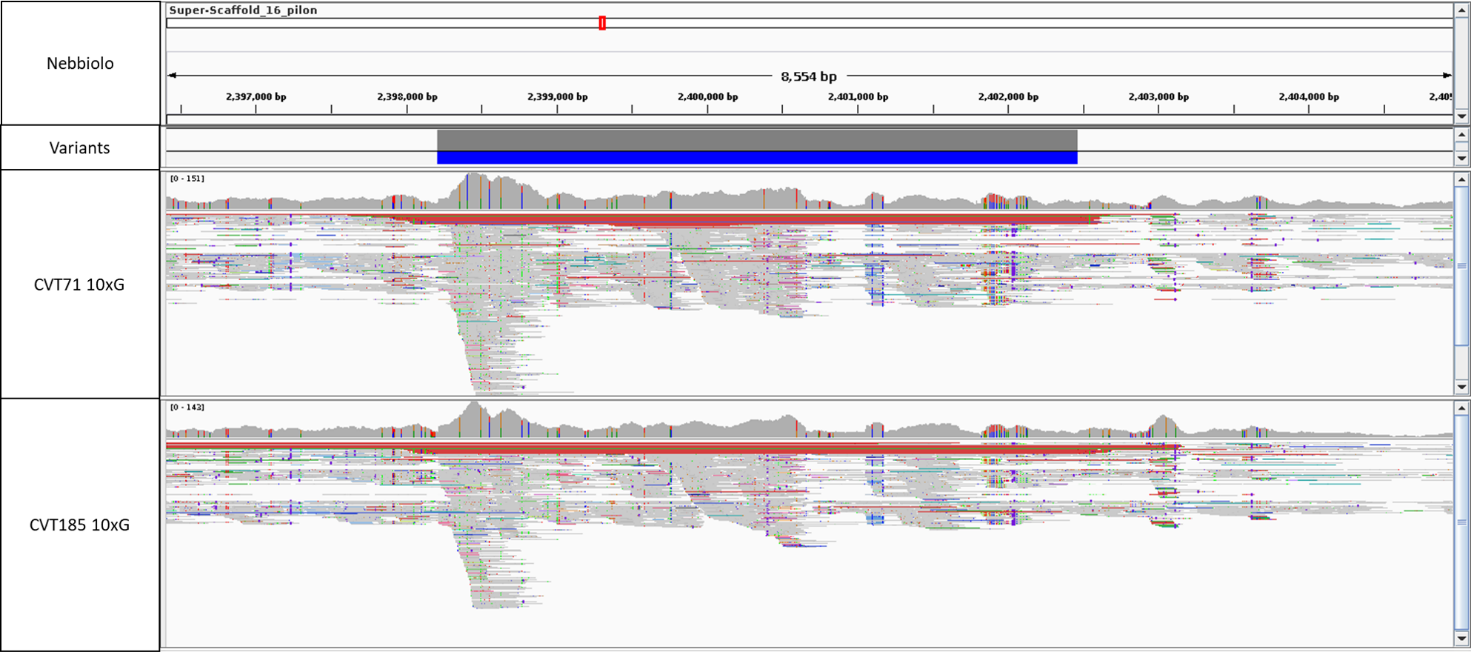


**Figure S5. IGV screenshot of a non-validated SV between ‘Nebbiolo’ clones CVT 71 and CVT 185.** In this example, the SV was called only for CVT 185 clone, but 10x Genomics reads support a heterozygous DEL also for CVT 71 clone.


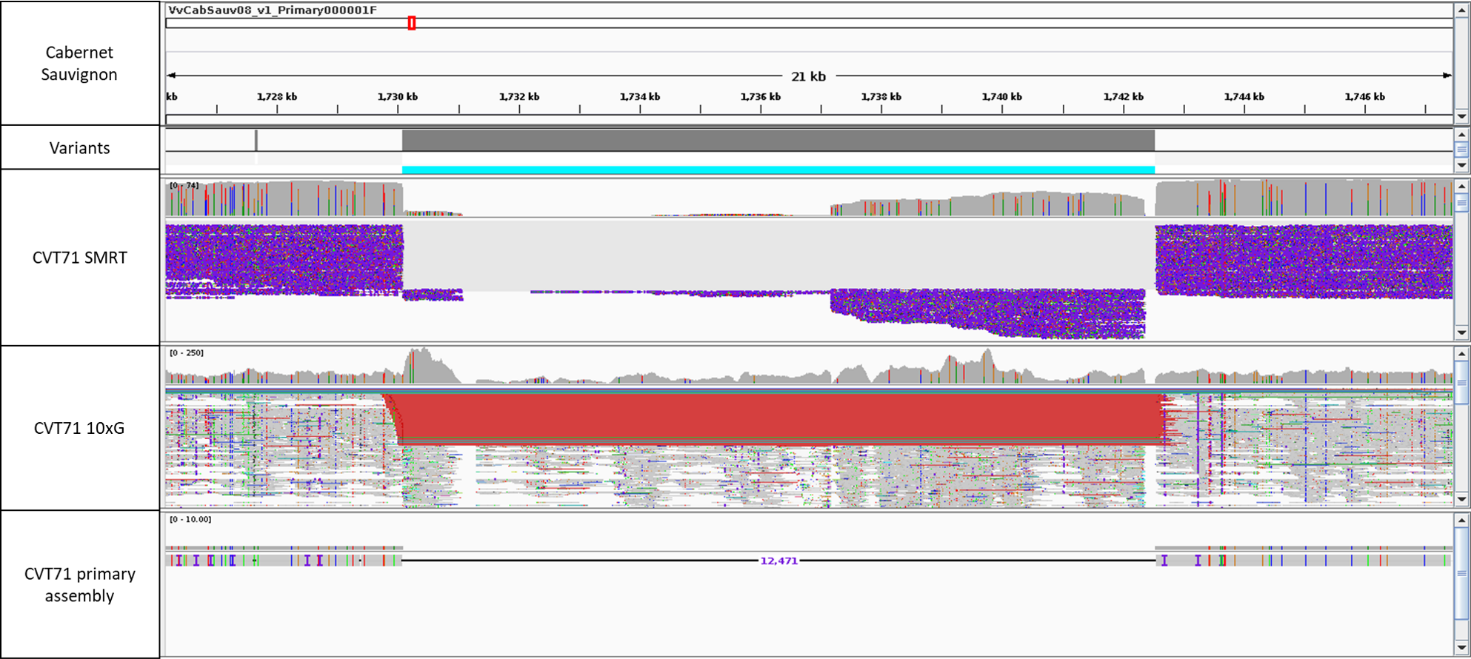


**Figure S6. IGV screenshot of a validated SV between ‘Nebbiolo’ and ‘Cabernet Sauvignon’.**

**Supplementary Tables**


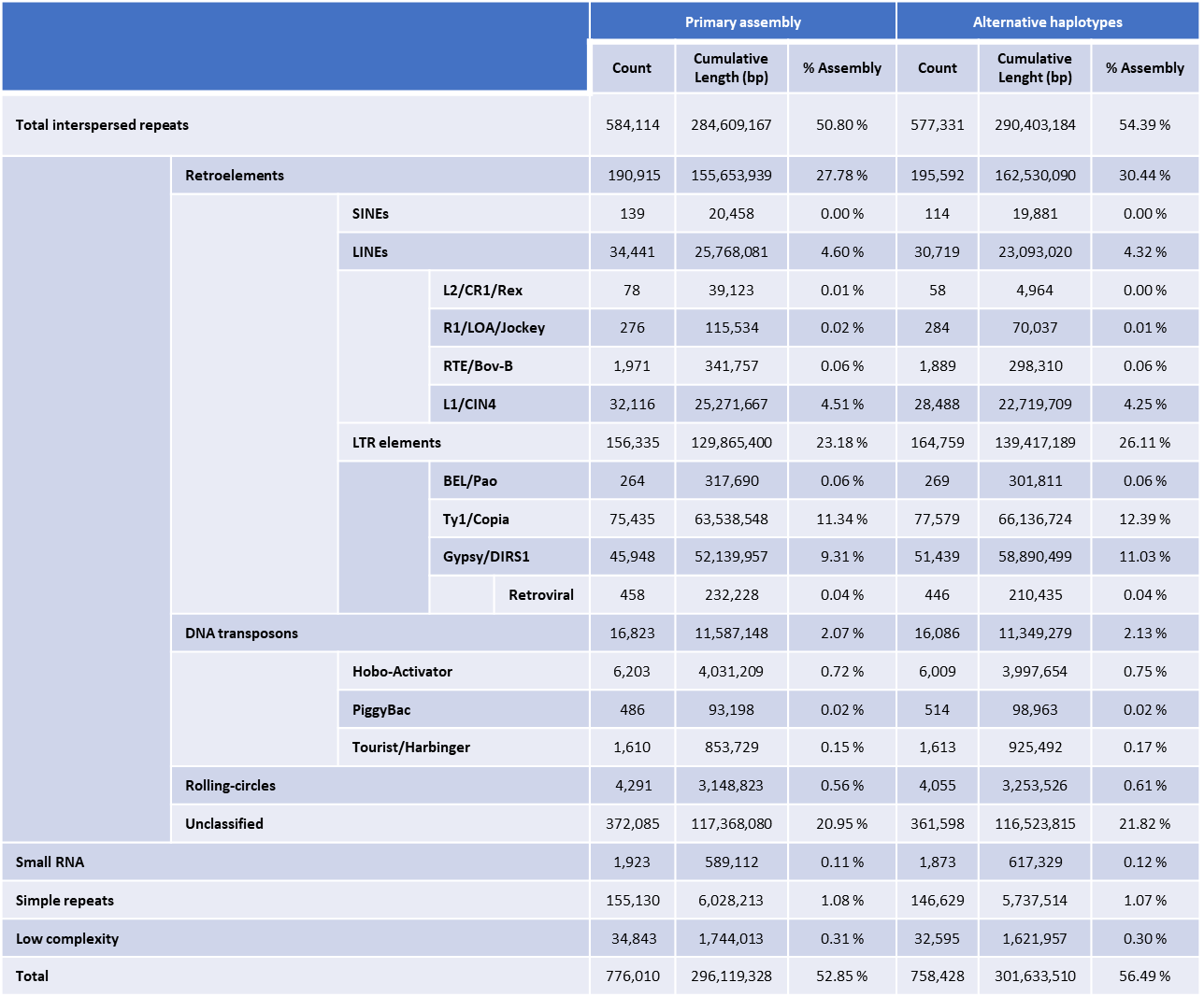
**Table S2. Repeats annotation of ‘Nebbiolo’ CVT 71 genome assembly.**

**Table S4. Details of the 20 SVs (deletions) picked for PCR validation. Detection method of each SV (PB = PacBio; 10xG = 10x Genomics and Genome = Genome to genome alignment), while LF = low frequency refers to SVs supported by the lower threshold established to consider an SV as true. Validation results refer to agarose gels shown in Figure S1. Validated means that both expected alleles were amplified, one allele means that only one of the two expected alleles was properly amplified. Only two SVs did not produce any PCR products.**

|  |  |  |  |  | **Expected amplicon lengths (bp)** | | **Final PCR conditions and electrophoresis** | | |  |
| --- | --- | --- | --- | --- | --- | --- | --- | --- | --- | --- |
| SV code  name | Detection method | SV  length (bp) | Average  Cov. | Reads supporting SV | Reference  allele | Deleted  Allele (SV) | Annealing Temp. | Extension time | Agarose gel | **Results**  **(Figure S1)** |
| PB_1 | PB | 208 | 55X | 37% | 1009 | 801 | 62 °C | 2' | 1% | Validated |
| PB_2 | PB | 225 | 85X | 26% | 725 | 500 | 64 °C | 1' | 1% | Validated |
| PB_3 | PB | 309 | 80X | 14% | 784 | 475 | 60 °C | 1' | 1% | Validated |
| PB_4 | PB | 199 | 105X | 31% | 569 | 370 | 60 °C | 1' | 1% | Validated |
| PB_5 | PB | 610 | 75X | 20% | 1283 | 673 | 60 °C | 1' | 1% | Validated |
| PB_LF_1 | PB | 81 | 2000X | 2% | 347 | 266 | 60 °C | 1' | 1.2% | One allele |
| PB_LF_2 | PB | 393 | 430X | 4% | 584 | 191 | 60 °C | 1' | 1% | Validated |
| PB_LF_3 | PB | 66 | 350X | 3% | 368 | 302 | 60 °C | 1' | 1.2% | Validated |
| PB_LF_4 | PB | 74 | 1100X | 2% | 538 | 464 | 60 °C | 1' | 1.2% | One allele |
| PB_LF_5 | PB | 65 | 1000X | 3% | 751 | 686 | Primers did not work | | | - |
| 10xG_1 | 10xG | 191 | 90X | 25% | 400 | 209 | 62 °C | 1' | 1% | Validated |
| 10xG_2 | 10xG | 193 | 80X | 19% | 541 | 348 | 60 °C | 1' | 1% | Validated |
| 10xG_3 | 10xG | 228 | 70X | 33% | 428 | 200 | 62 °C | 1' | 1% | Validated |
| 10xG_4 | 10xG | 875 | 50X | - | 1274 | 399 | 60 °C | 1' | 1% | Validated |
| 10xG_5 | 10xG | 281 | 90X | 31% | 662 | 381 | 64 °C | 1' | 1% | Validated |
| G_1 | Genome | 716 | 80X | 34% | 894 | 178 | 60 °C | 1' | 1% | Validated |
| G_2 | Genome | 684 | 70X | - | 1061 | 377 | 60 °C | 1' | 1% | One allele |
| G_3 | Genome | 551 | 80X | 33% | 1441 | 890 | Primers did not work | | | - |
| G_4 | Genome | 823 | 65X | 21% | 1102 | 279 | 60 °C | 1' | 1% | Validated |
| G_5 | Genome | 664 | 80X | 26% | 1128 | 464 | 60 °C | 2' | 1% | One allele |
|  |  |  |  |  |  | |  |  |  |  |


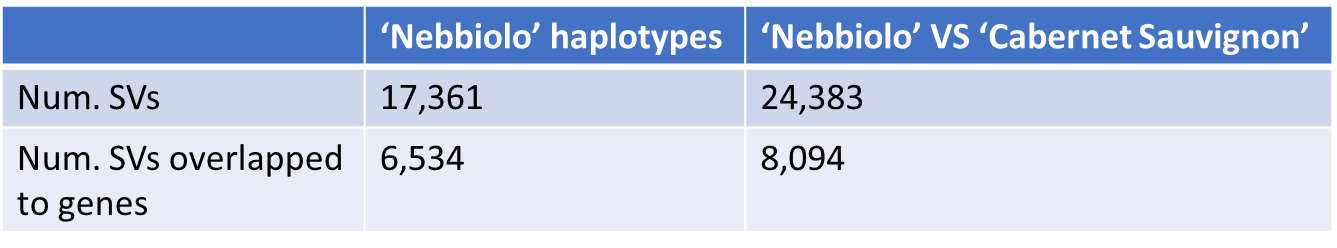
**Table S5. Total number of SVs and number of SVs overlapped to genes identified comparing ‘Nebbiolo’ haplotypes or ‘Nebbiolo’ to ‘Cabernet Sauvignon’.**

**Supplementary Methods**

**Validation of the bioinformatic approaches and thresholds to call heterozygous SVs:** We chose five SVs called with the three different strategies employed to call heterozygous SVs, namely reads alignment to the primary assembly (PacBio SMRT and 10xG) and genome to genome alignments (Genome). We also picked five SVs (called with SMRT reads) with Variant Allele Frequency < 5% as predicted by SMRT SV caller, which were also supported by our lower bound to consider an SV as true in visual inspection (i.e. four non-reference reads). The chosen SVs were deletions visually validated with IGV genome browser. In total, 20 PCR reactions were performed in 25 ul final volume reactions: Herculase II reaction Buffer (5x) 5 ul; dNTPs (25 mM) 0.25ul; Forward and Reverse Primers (10 uM) 0.625 ul each; Herculase II Fusion DNA Polymerase 0.25 ul; ddH2O (16.25 ul) and DNA (10ng) 2 ul. The general cycling conditions were: 95 ºC 3’; 35 cycles (95 ºC 30”; 60 ºC 30”; 72 ºC 1’) and 72 ºC 5’. For some SVs specific modifications in the cycling conditions (annealing temperatures and extension time) were introduced to optimize the amplification of the expected products (**Table S1**). For each reaction we were expecting to obtain two amplicons, one representing the reference allele without the deletion (longer fragment) and one non-reference allele containing the deletion (shorter fragment) (**Figure S1**).
